# Community-Based Livestock Breeding: Coordinated Action or Relational Process?

**DOI:** 10.3389/fvets.2021.613505

**Published:** 2021-05-24

**Authors:** Maria Wurzinger, Gustavo A. Gutiérrez, Johann Sölkner, Lorenz Probst

**Affiliations:** ^1^Faculty of Animal Sciences, Universidad Nacional Agraria La Molina, Lima, Peru; ^2^Division of Livestock Sciences, Department of Sustainable Agricultural Systems, University of Natural Resources and Life Sciences, Vienna, Austria; ^3^Department of Sustainable Agricultural Systems, Institute for Development Research, University of Natural Resources and Life Sciences, Vienna, Austria

**Keywords:** community-based breeding, livestock breeding, small-holder agriculture, multi-level perspective, breeding program

## Abstract

Over the past decade, community-based breeding programs (CBBPs) have been promoted as a viable approach to improving smallholder livelihoods through a systematic livestock breeding. CBBPs aim to initiate systematic breeding at the community level, including an organized animal identification and recording of performance and pedigree data. To ensure the breeding programs' continuity, building capacities, and ownership among participants are essential to the approach. This study's purpose was to understand how CBBPs have evolved in specific institutional settings and which dynamics occur in the course of implementation. We addressed these questions in reflective conversations with six coordinators of a diverse sample of CBBPs: goats (Malawi, Uganda, and Mexico), sheep (Ethiopia), alpaca (Peru), and cattle (Burkina Faso). The interviews and analysis were guided by categories of the multi-level perspective. The respondents considered lack of funding and weak institutionalization as the main constraints on the CBBPs. While the idea of participation and localized ownership was at the center of the programs, linear paradigms of knowledge transfer prevailed. In all cases, the impulse to start a CBBP came from individual researchers, who relied on intermediaries, such as extension agents, for implementation. Personal relations and trust were seen as both a factor in the success and a positive outcome of CBBPs. We conclude that these findings have different implications depending on how rural development is conceptualized: proponents of the innovation systems perspective would call for stakeholders to further align their interests and coordinate their actions. Proponents of process-relational concepts, in contrast, would not consider the CBBP a product but a starting-point for initiators and participants to continuously discover new ways of collaboration and engagement.

## Introduction

Community-based breeding programs (CBBP) have been promoted as a strategy for smallholder farmers to improve livestock breeds. Mueller et al. (1) described these programs as “typically related to low-input systems with farmers within geographical boundaries having a common interest to work together for the improvement of their genetic resources.” Typically, CBBPs define breeding objectives in a participatory process, which are then pursued in small-scale one or two-tier structures. The genetic resources are usually local so that CBBPs can also contribute to *in situ* conservation. Given the livestock keepers' role as the main agents in CBBPs, various authors have focused on their knowledge, needs, perceptions, and active participation (2–5). A wide range of literature also investigated the livestock keepers' selection criteria and breeding goals for different species and production systems (6–15). Using simulation models, another body of literature explored the potential genetic gains for diverse traits (16–20). Beyond these direct breeding-related questions, the effects of participating in a CBBP on economic benefits (e.g., marketing opportunities for breeding stock, meat, milk, and dairy products) to improve livelihoods were analyzed (21, 22). Opportunities for economic benefit largely depended on market access and integration, which were often poorly developed (23). Herold et al. (23) demonstrated, in their case study in Vietnam, how a pig breeding program could be strengthened via the integration of downstream processing and marketing stages.

As ultimate decision-makers, livestock keepers are usually considered the “owners of the breeding programs” in CBBPs. However, most initiatives also integrate different actors like extension services and research. Indeed, enabling policies, legal and institutional frameworks, and funding are seen as critical prerequisites to ensure the continuity of breeding programs (24–27). FAO (28) recognized in its Second *Report on the State of the World's Animal Genetic Resources for Food and Agriculture (SoW2*) that a diverse group of stakeholders is linked to breeding programs, suggesting the following categories: governments, breeders' associations or cooperatives, national or external commercial companies, NGOs or livestock keepers organized at the community level. Based on the report, Leroy et al. (29) concluded that development interventions should promote coordination among livestock keepers by creating and empowering cooperatives, associations, or community-based institutions. While CBBPs commonly start as small initiatives, the wish to scale out (including more farmers/communities in the region) and up (including additional actors, such as policymakers) is implicitly present. Kaumbata et al. (30) described the difficulties of CBBP scaling and concluded that it needs to be part of a breeding program's initial planning stage.

The question of how to initiate and facilitate change in agricultural practices is not specific to breeding, but a general concern in research and intervention to improve smallholder farmers' livelihoods. CBBPs and the strategies to mainstream the breeding approach in rural communities can be seen as part of this endeavor and emerged from participatory approaches to rural development (31). By including multiple stakeholders along the value chain and in the institutional environment, the approach also resonates with the more recent concepts of Agricultural Innovation Systems (32). The innovation systems perspective conceptualizes change in agricultural practice as emerging from the actors' interplay, strongly affected by the institutional environment (33). While particular aspects of CBBPs have been thoroughly analyzed (e.g., technical, financial), there has been no detailed discussion of the institutional and social dynamics that affect CBBP initiation, facilitation, and ownership transition. Therefore, this study aimed to understand how CBBPs evolve in specific institutional settings and which dynamics occur at the project level.

**Figure 1:**
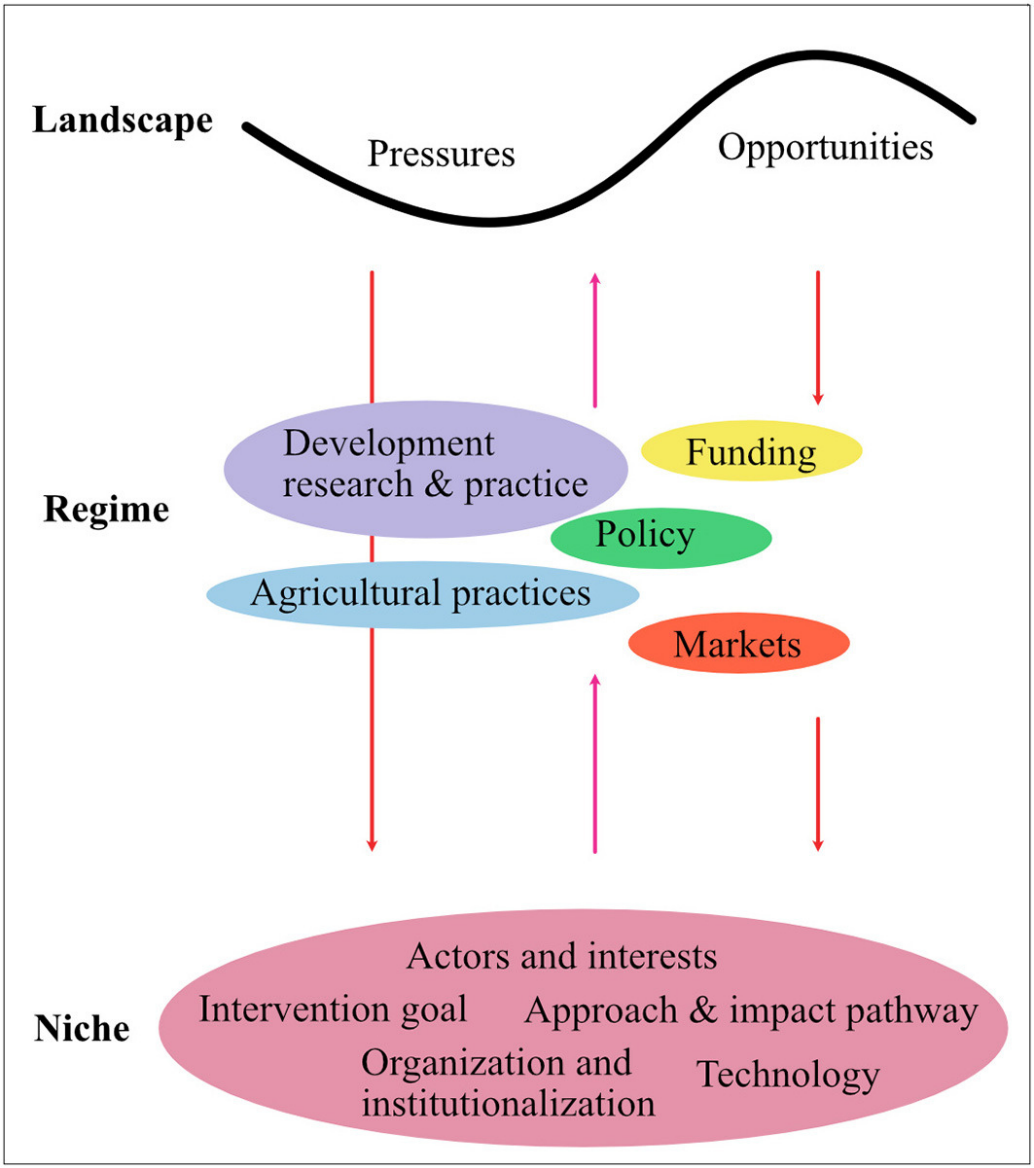
Analytical framework: CBBPs as niches linked to the regimes and landscape [Adapted from: El Bilali et al. (36)].

However, the perspective of innovation systems does not theorize processes at the group or personal level—including the values and meanings actors relate to their practice [e.g., (34, 35)]. Higher-level trends (e.g., climate or political dynamics) that can affect livestock breeding interventions are not easily integrated. To fully capture the evolution of different CBBPs, we, therefore, refer to El Bilali et al. (36) and their adaptation of the multi-level perspective (37, 38). We conceptualize CBBPs as *niches*, spaces where a novel approach to livestock breeding is introduced. This niche confronts or aligns with the *regime*, i.e., the current practices, rules, and institutions (e.g., agricultural policies, research in animal breeding, markets for livestock products). The *landscape* includes pressures and opportunities that cannot be influenced by niche actors but impact how the niche can develop. Examples of landscape trends are climate change, demographic change, and trade dynamics. The theoretical considerations were translated into an analytical framework specifying the categories and variables included in the data collection and analysis (Figure 1).

## Materials and Methods

### Research Instrument and Data Collection

The case studies were selected to cover a wide range of production systems and species (small ruminants, cattle, and alpaca). We included a mixed crop-livestock but also sole livestock production systems. The projects varied in initial size (number of participating livestock keepers), but were also at different implementation stages: the oldest project was initiated in 2009, whereas the most recent one was started in 2017. Table 1 shows the details of the included breeding programs.

**Table 1:** Details of selected community-based breeding programs.

**Country**	**Species**	**Implementation period**	**Funding sources**	**Involved actors**	**Interventions**
Malawi	Goats	2014–ongoing	USAID	• National University • Extension service • Farmers • International partners	• Male selection • Formation of cooperatives • Market linkages (butchers)
Uganda	Goats	2014–ongoing	USAID	• National research organization • Farmers • International partners	• Male selection • Formation of cooperatives • Market linkages • Animal health
Ethiopia	Sheep	2009–ongoing	Multiple	• National and regional research organizations • National University • International partners • Farmers	• Male selection • Formation of cooperatives • Market linkages • Animal health • Animal nutrition • Certification of breeding animals
Burkina Faso	Cattle	2017–ongoing	ADA	• National research organization • National universities • Extension service • Farmers • International partners	• Male selection • Formation of cooperatives • Animal health
Peru	Alpaca	2010–2020	VLIR	• National University • Farmers • International partner	• Male selection • Rangeland management
Mexico	Goats	2007–2015	Multiple	• National research organization • Farmers • International partners	• Male selection • Formation of cooperatives • Animal nutrition • Animal health

*USAID, United States Agency for International Development; ADA, Austrian Development Agency; VLIR, Flemish Inter-University Council*.

Since CBBPs are a niche also in animal breeding sciences, all coordinators of the considered CBBPs were personally known to the authors. We define “coordinator” as the person responsible for the design and for the implementation of the CBBP on the ground. The first author contacted the coordinators of the CBBPs, inviting them to participate in the study as expert respondents. All respondents were permanently employed researchers of universities or research organizations. An additional interview was carried out with a senior scientist who has an experience with implementing community-based breeding programs in different regions and was thus able to contribute more general insights.

The interviews, conducted via zoom or Skype in July and August 2020, followed a guide based on the analytical framework (Figure 1). The interview strategy was to facilitate an open conversation that creates a rich picture of the respondents' experiences with CBBPs.

All interviews were held in English and respondents gave free prior consent for the interviews to be recorded and analyzed.

### Data Analysis

The first author transcribed the interviews. For qualitative data analysis, we used a deductive coding strategy (Table 2) based on the analytical framework to structure the results. Atlas.ti Cloud was used for coding, which allowed all team members to work in parallel on the documents.

**Table 2:** Code categories and specification.

**Category**	**Codes**
Landscape	• Landscape drivers • Landscape constraints
Regime	• Policies • Markets • Research and education paradigms • Default breeding paradigms • Funding
Niche	• Project goal and impact pathway • Organization & institutionalization • Actors & interests • Technology • Character of activities
General reflection	• Male selection • Formation of cooperatives • Animal health

## Results

We report the results along the analytical framework, starting with the higher-level trends at the landscape level, then narrowing the focus on the regime and niche.

### Landscape Level: Funding and Population Dynamics

The respondents did not explicitly refer to the higher-level drivers and constraints in their reflection on their respective CBBP programs' history. They considered funding as the primary external variable they could not influence, which directly impacted their efforts' effectiveness and permanence.

“*If you don't have money, you don't have a project. If you don't have a project, you cannot work on anything.” (Respondent 2, Mexico)*

While all CBBPs had started as externally funded projects, the respondents agreed that a shift toward continued national support would be necessary for community-based breeding to be successful. CBBPs cannot be considered a one-time intervention:

“*When people say ”Yes, let's do CBBP,” I say, do you have plans to invest over a long period into this program? If you don't want to do that, and you see it as a short term—forget it. It is not worth starting, it is something where you waste your money, you need long-term funding, and you need the support from the national system to do for a long time.” (Respondent 3, Ethiopia)*

In addition to funding, the role of the policies aimed at conserving and improving local animal genetic resources was also emphasized. Such policies provide the legal framework for the implementation of the breeding programs.

“*And this has to be backed up by policies. A national policy saying the improvement and management of the national animal genetic resources of the country.” (Respondent 7, Bolivia)*

Beyond the political landscape, the respondents also acknowledged that broader societal dynamics could drive or constrain a change of breeding practices. In the case of Bolivia, for example, the aging rural population, outmigration of the young labor force, and small farming units were considered as factors that limited continuous breeding efforts. However, in turn, low productivity and vulnerable livelihood systems may also inspire efforts to introduce alternative livestock breeding approaches.

### Regime: Transfer of Technology and Participatory Approaches

Current livestock breeding practices in the analyzed cases involved different species but were commonly characterized by low levels of systematic breeding, which includes animal identification and recording of performance and pedigree. In free grazing arrangements, random mating was the default practice, and particularly in meat-oriented systems, negative selection due to selling of the best young males was a significant challenge. This practice resulted in a shortage of locally available breeding males. Also, the prolonged use and the rare exchange of breeding males led to the perceived high inbreeding levels. Where deliberate breeding efforts were made, criteria were not consistently applied nor the records kept. Against this background, according to the respondents, the general perception was that performance improvement would require the introduction of exotic breeds and crossbreeding:

“*When they say that we bring in a goat project, they expect something to be introduced to their system. And that something should not be local, but exotic. So, that was a major drawback to the CBBP.” (Respondent 4, Malawi)*

However, the lack of adaptation of exotic breeds, loss of breed diversity, and lack of infrastructure and funding caused the systematic crossbreeding schemes to be unsuccessful in most cases. Consequently, it became a general assumption in development programs that systematic breeding in low-input systems with smallholders was not a promising strategy.

This tension was also reflected in the way the respondents conceptualized their own efforts in facilitating a community-based breeding. Their approaches reflected different paradigms, often simultaneously in a single project. Fundamentally, all analyzed CBBP initiatives were part of the donor-driven, project-based development logic. Most respondents also referred to institutions from “outside” (universities from the global North, CGIAR-centers) as essential in the start-up phase of the CBBPs. When reflecting on the specific projects, the idea of transferring the approach of breeding through CBBPs from the researcher through the extension to livestock keeper emerged frequently. Also, the question of whether a CBBP is a social intervention or needs to be run by a breeding scientist arose:

“*For example, in Mexico, we had a colleague who is technically very solid, but he says that a CBBP is just talking, just sociology, this is not animal breeding.” (Respondent 9, Mexico)*

At the same time, all respondents considered their CBBP as highly participatory and suggested that their role was mainly on guiding the participants. Even in this participatory narrative, however, the livestock keepers' ownership in the projects seemed to be limited. In almost all the cases, the CBBPs were wholly dependent on the initiators for keeping the momentum, and participants often expected the projects to “bring” something immediately valuable to them.

Although the policy level was considered important by the respondents, the CBBPs were not explicitly constrained or strengthened by the national livestock policies. The projects made an effort to legitimize the approach toward the policymakers, who were generally supportive mainly on where funding was brought in, and successes were visible and could support their agenda. In most projects, the respective ministries were directly involved—in Uganda, the implementing body was a parastatal institution directly under the ministry, and the other projects consulted with ministry representatives in the site selection and gave progress reports. It is only in Peru where no formal exchange with the policy level was established.

According to the respondents, an aspect that had been frequently overlooked in breeding-related projects was the market linkages. For CBBPs to take lasting roots, securing market access for their products (meat, fiber, milk) and, in a further step, breeding animals are essential. CBBP initiatives can play a facilitating role in establishing market linkages.

“*You have a breeding program, but it needs to be embedded in the wider context if you want to have this value chain transformation of the livestock sector. Because having the better animals alone, but you also need a market that will take these improved animals.” (Respondent 3, Ethiopia)*

### Niche: Projects to Improve Livelihoods Through Community-Based Breeding

The CBBP initiatives had the common long-term objective of improving the livestock keepers' livelihoods. In the medium term, the projects hoped to achieve improved livestock breeding practices and, consequently, higher productivity at the community level.

“*You ask if you can live off the products of 30 llamas? Can I provide my livelihood? How much would I have to improve my llamas in order to make my living?” (Respondent 7, Bolivia)*

The assumed impact pathways followed a linear logic, proposing to scale-out CBBP practices through extension or NGO actors while simultaneously scaling-up the CBBP approach at the national and local policy levels. The central user outcome was to build the livestock keepers' capacities in systematic breeding for genetic improvement, and in some cases, supporting institutionalization. The marketing of animal products or breeding animals (livestock trade, dairy sector, butchers) was not typically included but considered relevant when looking back at the CBBP experiences. Policymakers at different levels, from national actors to local administrative units, were provided with evidence on the potential of CBBPs and explicitly addressed to mobilize further support for the initiatives.

In a typical CBBP arrangement, researchers calculated a ranking of the potential breeding males based on the data collected by the enumerators, who were often extension agents. The collection and management of data was a challenge in all projects, and in Ethiopia, the use of tablets was a significant improvement. The ranking was provided to the livestock keepers' selection committees, who made the final selection based on the ranking and their own preferences. The respondents considered this final step as the central aspect of signaling CBBP ownership to the livestock keepers.

Except for Mexico, all projects focused initially on the implementation of breeding programs. In Mexico, the CBBP emerged from a project on nutrition and animal health interventions. The other projects later included accompanying activities and outcomes (e.g., rangeland management plans, vaccination, and animal health checks) to bridge the time lag between breeding efforts and visible results.

The impulse to start a CBBP came in all cases from individuals at universities or research organizations who had personal ties to a specific region. Except for Peru, these initiatives could not build on existing associations or cooperatives, but all respondents saw such institutions as necessary to start a CBBP and ensure its continuity effectively. The respondents further stressed the importance of institutionalization:

“*What we underestimated was the institutional set-up, which is really needed. How much institutional set up you actually need and how well this has to be set up.” (Respondent 3, Ethiopia)*

In some cases, respondents found that livestock keepers were less interested in collaboration than expected, or livestock was not their focal activity. Where the projects facilitated setting up of cooperatives or associations, the collaboration with the project was not specified in formal agreements. In all cases, a crucial role was assigned to intermediaries, such as extension agents, who were counted on to link the research system (national and international universities and institutions) to the livestock keeping communities, record data, and monitor breeding implementation. In Peru, however, an extension system was not in place, and partly, the projects had no choice but to pay the extension agents—which may, in turn, might give rise to problems of continuity:

“*They consider [the CBBP] their own. They are government employees, so you can ensure long-term sustainability. In other regions, when we sent them some money, this is how they paid the enumerators. This is not the right way to do.” (Respondent 4, Ethiopia)*.

Combining the CBBP project with the capacity building in higher education (involving MSc/PhD candidates, technical staff) was evaluated as a very positive outcome by the respondents. Some universities adapted their curricula as a result of their participation:

“*And we have already got two courses. One is animal breeding and genetics at the undergraduate and a similar one applied animal breeding at the Masters level. We have integrated this and we got another course called ”Farm animal genetic resource management” and part of the conservation methods, which is heavily related to goat breeding. The concept of community-based conservation has come on board. So, we are now using these as case studies.” (Respondent 3, Malawi)*

Contingent upon the projects' capacities, scaling-out to neighboring communities and scaling-up through including additional actors were common strategies. Out-scaling did not always follow a planned process, but neighboring livestock keepers could get an idea of the success in informal contacts. Up-scaling proved to be difficult in some cases because organizations identified as potential partners did not have the necessary technical know-how and the required budget to get involved.

By bringing together actors along the value chain and the wider innovation system, the CBBPs resonated with the current approaches of multi-stakeholder platforms. Within the stakeholder groups, specific inspiring individuals had a pivotal importance in driving the CBBPs—be it at the research, policy, extension, or farm level. At the same time, the data show that agency in the initiatives was concentrated around the initiating researchers and practitioners—who described their involvement mainly using verbs like monitor, use, show, start, make, work, and move. Participants, on the other hand, were referred to mainly using passive forms, such as: were taught, were informed, were trained, and were requested. Accordingly, the respondents described success on the participants' side using attributes such as improved understanding, new abilities, or recognizing change.

Nevertheless, when reflecting on the key factors of success, several respondents strongly emphasized the importance of *being* with the livestock keepers, of relating in a trustful and committed way:

“*And it was part of having a huge lunch over there with enchiladas, tacos and much good stuff for food and some music. It was kind of a party.” (Respondent 9, Mexico)*

The dilemma of initiating a process that should be owned by someone else thus remained unsolved. Entrusting livestock keepers with more responsibility right from the beginning and giving them more decision-power was seen as one way to foster ownership:

“*Start and let them lead more the program. Let that they organize, that they make some organizations, that could be among them in order to strengthen the alpaca breeding program.“ (Respondent 1, Peru)*

### Reflections: How to Make Community-Based Breeding a Success

When reflecting on further support that would have helped the CBBPs take firmer roots, the respondents mentioned a stronger and continuous backing at the national and local policy level. The role of intermediaries in facilitating the introduction of CBBPs was described as crucial where extension services were in place—the lack of such facilitation was, in turn, seen as a major constraint. This constraint was related to the institutionalization and social momentum necessary to establish or strengthen breeding associations who would own the CBBP after the end of a project. Respondents saw these institutions as essential to fostering the trust necessary for exchanging animals. At the implementation level, the respondents highlighted that appropriate tools (e.g., offline-ready apps) could make a significant difference in the daily work of a CBBP.

Reflecting on the CBBP process, the respondents described several tensions and ambiguities that a project has to navigate in the different phases from inception to hand-over. First, all respondents saw a need to better understand the values, knowledge, and livelihood strategies of the potential CBBP participants before introducing the concept. To gain such understanding and to build trust and a good working relationship, the respondents considered it essential to explicitly invest in continuous communication, transparency, and timely feedback. However, winning trust takes time and requires consistent action and tangible results:

“*Farmers just trust you when they see what you are saying is right. So I think, in areas where we have been, we were quite transparent and we tried to support them and they see something really happening on the ground.” (Respondent 3, Ethiopia)*

At the same time, CBBPs require capacities that participants may need to develop. All experts agreed that capacity development, not only for the livestock keepers but also for the technical staff, was an essential element of their projects. Second, better tools to register animals, record herd development, and certify breeding animals would ease the implementation. However, providing these services may jeopardize participant ownership and commitment. Third, CBBPs are long-term investments that require continuity, particularly at the facilitation and management level. This, however, does not fit well with the project-logic in research for development. Finally, institutionalizing CBBPs at a community level and beyond proved to be essential. Such institutions, however, cannot be imposed and need to balance the structural requirements of a CBBP with the freedom for participants to take ownership and initiative beyond the project.

## Discussion

### CBBPs Are an Established Niche

Our results support the current perception in the literature: CBBPs are an established niche—approach to livestock breeding in smallholder agriculture, with the potential to improve livelihoods (21, 22). The number of publications related to community-based breeding has increased over the past several years [e.g., (27, 28)]. The universities which partnered in the studied cases are examples of how the approach is transmitted rather quickly into specific courses and can later be formally integrated into the entire curricula. This integration adds to the legitimacy of CBBPs, and future graduates may accept and apply the approach more readily. Our results also suggest that CBBPs have not reached a mainstream practice stage, embedded in the rules and institutions at a regime level. If we consider community-based breeding as a viable pathway to improve livelihoods, the question arises of how a more substantial change of livestock management and breeding could come about. We discuss this question from two different perspectives: coordinated action in an innovation system and self-organization in flexible social relations.

### Coordinated Action Toward Community-Based Breeding

With their CBBPS, the respondents met an institutional environment that lacked organization or favored the common transfer-of-technology approaches. As a response, all respondents called for a better organization and institutionalization of livestock breeding, including CBBP mechanisms, in their respective project areas. According to Picot et al. (39), the institutional term ”organization“ covers a whole system of institutions like markets, agreements between business partners, but also the legal framework, and public organizations. Indeed, Herold et al. (23) proposed that ”organization is an important factor in animal breeding.“ The authors distinguish between the process-oriented, instrumental, and institutional definition of ”organization.“ The main focus in the projects we investigated for this study was at the level of “process-orientation.” Data recording and performance testing of selection candidates, an area for which much time and effort was spent, was a typical example. Roles and responsibilities for the different steps were coordinated and shared between livestock keepers, field staff, and researchers. The ”instrumental“ dimension refers to a breeding organization's internal structure, in our case, livestock keepers' cooperatives or associations. This structure encompasses the rules and decision-making mechanisms of these organizations. Although the respondents repeatedly emphasized the importance of the cooperatives, they also indicated that knowledge about facilitating institutional change was limited among the initiators of the CBBPs. In general, the literature suggests that livestock keepers can benefit from being a cooperative member, but membership can come with problems and pitfalls. In the European context, Schmitt and Momm (40) recommended a two-level organizational structure for breeding associations with a general assembly for all members and a board consisting of elected representatives. To our knowledge, this issue has not been addressed in the context of smallholder farming, thus being an area of research that should be given more attention in the future. Several authors (1, 16, 19, 20) discussed different breeding strategies such as central vs. dispersed nucleus or group breeding systems, but their analysis does not address the question of how these different approaches should be reflected in the structure of breeding organizations.

Beyond the organization of breeding, a further point of discussion both among the respondents and in the literature is the vertical integration of breeding associations in the value chain. This could create opportunities for members by adding value to primary products. Herold et al. (41) illustrated how such integration could be achieved in a Vietnam pig breeding program. In our study, respondents also suggested that a division of labor between specialized breeders and regular livestock keepers as their customers could be a future scenario.

Finally, the question of funding and continuity emerged as the primary concern of respondents. All presented cases had started as externally funded projects but without a clear vision of how the breeding programs should be financed in the long-run. The initiators seemed to have assumed that the national or regional government would take up this role. However, after 10 years of continuous effort, the sheep breeding program in Ethiopia was still partly dependent on external funding, even though there is a strong political interest from the national government. CBBPs are included in the livestock development plan as the breeding strategy of choice. Lobo (25) and Gowane et al. (26) stressed the importance of public funding and the challenges caused by an insufficient and fluctuating support. Accordingly, they proposed to develop breeding programs that are self-sustainable and profitable. Where a private sector is not well-developed or even absent, this may be very ambitious.

In conclusion, coordinated action and alignment of interests are imperative to promote CBBPs from the innovation systems perspective. From the outset of community-based breeding programs, the understanding of the stakeholder network and institutional environment needs to be a primary focus—as well as the facilitation of institutional learning and creation of ownership.

### Community-Based Breeding as a Relational Process

What if it is impossible to meaningfully describe and replicate an institutional set-up that will allow the scaling of CBBPs? What if there is no continuity in the collective action without the initiating researcher? These questions, resonating with the ambiguities and tensions we identified in the respondents' reflections, arose during this study's write-up.

From an innovation systems perspective, we discussed coordinated action and alignment of interests as imperative. In the data, however, there is little evidence of CBBPs being a stable systemic arrangement, even in the most structurally established case of Ethiopia. Instead, the analyzed CBBPs seem to be constantly evolving, and discontinuation is not an unlikely scenario. The main commonality we found across the cases was the impulse of an “intentional and purposeful activity” (42)—driven by researchers who shared the belief in improving livelihoods locally, in a fair and participatory manner. At the same time, the different CBBPs remained fragmented, as unequal power relations prevailed with researchers and extension officers being in the position of the key mediators. We also have to assume that the communities and breeding associations involved were not necessarily egalitarian, but highly differentiated—an aspect that did not come up at all in the respondents' reflections. Most tangible were the fragmentations when respondents described their efforts to reconcile project logic and collective action, steering and letting go of their program, and being an expert on breeding but trying not to impose this knowledge on the participants.

This confusion cleared when the respondents reflected on what worked well: the integration of community-based breeding in their own teaching practice at the University, the time they spent celebrating in the communities, the trust that developed between them and the livestock keepers, and the personal satisfaction derived from seeing community-based breeding in action. This finding is consistent with Umans and Arce (43), who suggested that change is more likely to be the outcome of engaging with the reality than of planning and design. Indeed, it has been disputed whether collective action, institutions, and social norms can be planned at all (44).

Accordingly, we could argue that the absence of institutions allowed the initiators to create CBBP interactions in a way they value. Instead of focusing on institutions that enable or constrain, and seeing a CBBP as an end-product separate from the researcher, this perspective would consider the CBBP as an ongoing process in which relations between social actors are made, transformed, and abandoned (45). Process-relational theories propose that the order in institutions is contingent, not continuous—the only social reality would be the series of events and relations that temporarily create something called CBBP. Consequently, the CBBP would be something very diverse for the plurality of the people involved.

The process-relational perspective does not resonate well with our wish for clear causalities and stability—it does, however, provide openings for new conceptualizations of how CBBPs could be seen and promoted: as a practice that the initiators coherently integrate into their work and lives (42). When community-based breeding becomes part of their own continuous engagement as members of the community, social change may be more likely to emerge from a sense of responsibility and accountability.

## Conclusion

Community-based breeding programs have been promoted as a viable approach to systematic livestock breeding in low-input smallholder farming contexts. The purpose of this study was to understand how CBBPs evolve in specific institutional settings, and which dynamics occur at the project level. The respondents considered funding as the primary higher-level variable, which they could not influence. While the idea of participation and localized ownership was at the center of CBBPs, the programs had to follow a typical project logic as researchers remained the main mediators, and linear paradigms of knowledge transfer prevailed. Most CBBPs sought to lobby for policy support, and some included efforts of market integration—an aspect that had been frequently overlooked in the past. In all cases, the impulse to start a CBBP came from individual researchers, who relied on intermediaries, such as extension agents, to implement the program. Relating in a trustful and committed way was seen as a critical outcome and success factor, while further institutionalization was called for. We conclude that CBBPs are an established niche concept—to support social change toward systematic breeding in smallholder contexts, two different perspectives may be helpful: from an innovation systems perspective, coordinated action and alignment of interests would be necessary. From the perspective of process-relational concepts, CBBPs could become a part of the researchers' daily practice and their continuous engagement with a community.

## Data Availability Statement

The raw data supporting the conclusions of this article will be made available by the authors, without undue reservation.

## Ethics Statement

Ethical review and approval was not required for the study on human participants in accordance with the local legislation and institutional requirements. The patients/participants provided their written informed consent to participate in this study.

## Author Contributions

MW and LP developed the concept, analyzed the data, and prepared the first draft of the manuscript. GG and JS commented on the manuscript. All authors read and approved the final draft.

## Conflict of Interest

The authors declare that the research was conducted in the absence of any commercial or financial relationships that could be construed as a potential conflict of interest.
